# Velamentous Cord Insertion With Vaginal Delivery: A Case Report and Review of the Literature

**DOI:** 10.7759/cureus.101487

**Published:** 2026-01-13

**Authors:** George Mpourazanis, Stefanos Flindris, Konstadinos Pantazis, Dimitrios Alefragkis, Petros Papalexis, Ioannis Korkontzelos, Ioannis Kosmas, Apostolos Ntanasis, Pietro Serra, Antonio Simone Laganà, Rüdiger Schulz-Wendtland, Panagiotis Tsirkas

**Affiliations:** 1 Department of Obstetrics and Gynecology, General Hospital of Ioannina "G. Hatzikosta", Ioannina, GRC; 2 Second Department of Obstetrics and Gynecology, Hippokration Hospital of Thessaloniki, Thessaloniki, GRC; 3 Unit of Urogynecology, Second Department of Obstetrics &amp; Gynecology, Aristotle University of Thessaloniki, Hippokration General Hospital, Thessaloniki, GRC; 4 Fourth University Department of Internal Medicine, Unit of Hematology and Oncology, University General Hospital Attikon, Chaidari, GRC; 5 School of Health Sciences, Department of Nursing, National and Kapodistrian University of Athens, Athens, GRC; 6 Unit of Endocrinology, First Department of Internal Medicine, Laiko General Hospital, National and Kapodistrian University of Athens, Athens, GRC; 7 Department of Anesthesiology, General Hospital of Ioannina "G. Hatzikosta", Ioannina, GRC; 8 Unit of Obstetrics and Gynecology, Paolo Giaccone University Hospital, Palermo, ITA; 9 Department of Health Promotion, Mother and Child Care, Internal Medicine and Medical Specialties (PROMISE), University of Palermo, Palermo, ITA; 10 Unit of Gynecologic Oncology, ARNAS "Civico-Di Cristina-Benfratelli" Department of Health Promotion, Mother and Child Care, Internal Medicine and Medical Specialties (PROMISE), University of Palermo, Palermo, ITA; 11 Department of Gynecology and Obstetrics, University Breast Center for Franconia, University Hospital Erlangen, Friedrich-Alexander University of Erlangen-Nuremberg, Erlangen, DEU

**Keywords:** cesarean delivery, cord insertion, vaginal delivery, vci, velamentous cord insertion

## Abstract

Velamentous cord insertion (VCI) is a rare placental abnormality in which the umbilical cord enters the chorionic membranes instead of the placenta, leaving the umbilical vessels unprotected. This pathological condition carries an increased risk of adverse perinatal outcomes, such as fetal growth restriction, preterm delivery, abnormal fetal heart rate patterns during labor, and perinatal morbidity, which often lead healthcare professionals to choose cesarean section to reduce postnatal risks. Early prenatal diagnosis using color Doppler imaging and comprehensive ultrasound is of utmost importance for proper risk assessment and birth planning. This case study presents a 28-year-old woman in whom screening for abnormalities during the second trimester of pregnancy detected a prenatally detected velamentous umbilical cord insertion. The pregnancy was closely monitored, and the patient chose vaginal delivery despite the multidisciplinary team's recommendations for a cesarean section, with continuous intrauterine monitoring of the fetus. At 38 weeks of gestation, a successful vaginal delivery was performed with vacuum assistance, without complications for the pregnant woman or the newborn. A postnatal examination of the placenta revealed velamentous attachment of the umbilical cord, and both the mother and the newborn recovered without complications. In pregnancies complicated by velamentous umbilical cord insertion, vaginal delivery may be a safe and feasible option when accompanied by appropriate prenatal evaluation and continuous intrauterine monitoring. These findings are supported by other case studies following a comprehensive review of the existing literature. Instead of preferring cesarean section, individualized obstetric care can improve outcomes while respecting the mother's preferences. However, further extensive research is needed to develop evidence-based guidelines on the best methods of delivery for such pregnancies.

## Introduction

Velamentous cord insertion (VCI) occurs when the umbilical cord attaches to the chorionic membranes, instead of a safer location in the central or peripheral area of the placenta. This abnormal attachment results in a lack of the mechanical support normally provided by Wharton's jelly, which normally protects the umbilical arteries. This anatomical vulnerability poses significant risks to fetal health because it increases the chances of vascular compression, stretching, or rupture. These risks are particularly acute during uterine contractions, labor, or when the membranes rupture [1,2].

VCI is a condition that results from abnormal placental implantation and vascular development during the early stages of pregnancy. Findings from histopathological studies show that the fetal membranes are usually penetrated by long, helical umbilical arteries, which are often located at a considerable distance from the placenta. This anatomical arrangement makes the membranous veins particularly sensitive to external mechanical pressure, leading to possible complications, such as disruption of fetal blood flow, severe fetal hypoxia, or significant hemorrhage, especially in cases where VCI is associated with vasa previa. Although abnormal vascular branching is a common finding, the structure of the placental disc generally remains intact, highlighting a complex interaction between vascular abnormalities and the overall integrity of the placenta. This condition poses serious risks to fetal health, highlighting the need for careful monitoring and management of affected pregnancies [3,4].

According to recent reports, the incidence of VCI in singleton pregnancies ranges from approximately 0.4%-2.4%. However, this condition is more common in multiple pregnancies and pregnancies using assisted reproductive technologies, suggesting a significant risk factor associated with these types of pregnancies [5]. The clinical findings of VCI are associated with a number of adverse perinatal outcomes, such as preterm birth, low birth weight, small-for-gestational-age newborns, abnormal fetal heart rate patterns during labor, and increased rates of operative vaginal deliveries and cesarean sections [6].

This report presents the case of a 28-year-old woman with a known history of VCI, highlighting its successful management through vaginal delivery. The report aims to help healthcare professionals make informed clinical decisions regarding delivery strategies in pregnancies complicated by VCI. In addition, it includes an overview of the pathological characteristics associated with VCI and discusses the relevant recent literature on the subject, providing a comprehensive understanding of the implications of the condition for obstetric practice.

## Case presentation

**Figure 1:**
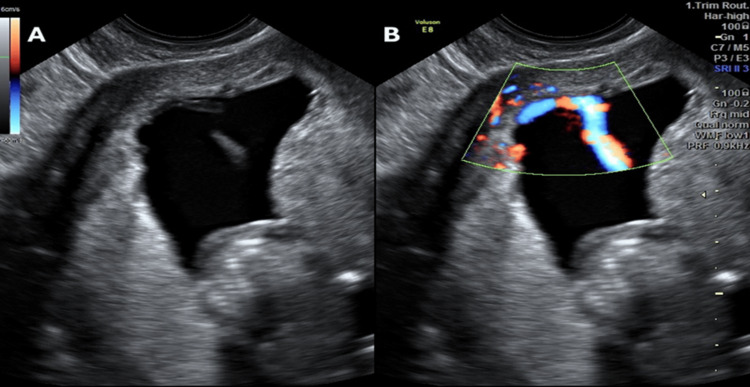
(A) The cord vessels running through the membranes before reaching the placenta, appearing as echogenic linear structures distinct from the placental tissue. (B) Using color Doppler, emphasizing the **abnormal pathway of the umbilical vessels**—visualized as red and blue color signals—spreading unprotected through the membranes. The two ultrasound images illustrate a case of velamentous cord insertion. In this condition, the umbilical cord attaches to the fetal membranes (chorioamniotic membranes) instead of connecting directly to the placental mass. These vessels lack Wharton’s jelly, rendering them susceptible to compression or rupture, especially during labor. The observations are typical of a velamentous cord insertion, a critical prenatal diagnosis due to associated risks, such as fetal growth restriction, vasa previa, and complications during labor.

We describe the case of a 28-year-old woman with a known history of VCI detected and diagnosed via fetal Doppler at the 20-week gestation full anomaly ultrasound scan (Figure 1). 

At 38 weeks of gestation, she arrived at the obstetrics emergency department with regular uterine contractions, which commenced two hours prior to her abdominal pain, and 4 cm cervical dilatation on obstetrical vaginal examination. Upon admission, the patient's vital signs were recorded as blood pressure at 130/75 mmHg, a temperature of 37°C, a pulse rate of 82 beats per minute, a respiratory rate of 19 breaths per minute, and an oxygen saturation level of 98%. She had no prior pregnancies, parity, abortions, miscarriages or use of assisted reproductive technology, according to her obstetric medical history. The woman conceived naturally. Her body mass index (BMI) was reported to be normal at 23.9 kg/m².

**Figure 2:**
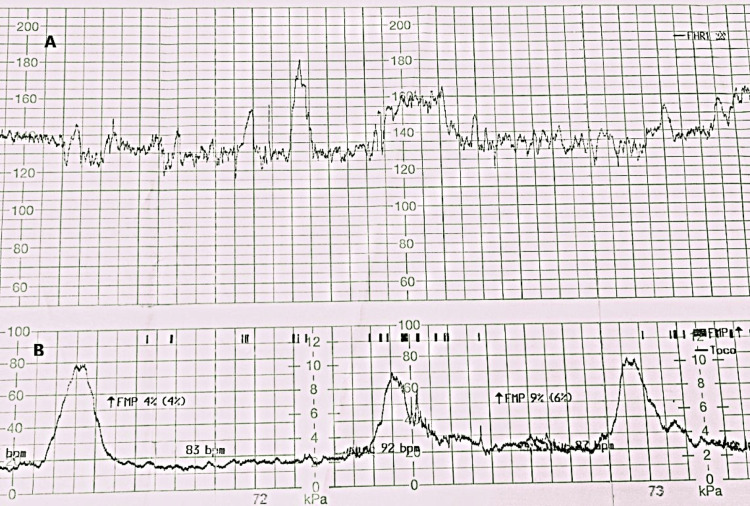
(A) Normal baseline fetal heart rate (FHR) of 130–140 bits per minute (bpm) with significant variability and frequent accelerations, indicating healthy autonomic nervous system function and adequate fetal oxygenation. (B) Illustrating the link between uterine activity and fetal movement, noting that fetal motions are associated with FHR accelerations, leading to a reactive non-stress test (NST) during labor.

The patient disclosed a medical history of gestational diabetes mellitus, which was treated by moderate exercise and diet. The pregnant woman during the first trimester of pregnancy showed TSH hormone levels of 3.7 mIU/L (0.1-2.5), so she was diagnosed with gestational hypothyroidism based on the relevant guidelines, and therefore treatment with levothyroxine T4 50 mcg 1x1 per day was initiated [7]. One month after starting levothyroxine treatment, thyroid-stimulating hormone (TSH) levels returned to normal levels for gestational age. Her gynecological history indicates that menarche started at age 15, with menstrual cycles occurring every 30 days and lasting four to seven days. She indicated no known allergies, use of alcohol, or smoking habits and reported no previous surgeries. A Pap smear performed a year prior yielded a negative result for malignancy. She was urgently admitted for intrapartum care, fetal monitoring, and possible vaginal delivery management. When she reached the labor ward (delivery suite) due to her intense and regular contractions, a repeat obstetric vaginal exam was performed. The cervical dilation evaluated by vaginal exam was estimated at 7 cm, and on consultation and delivery plan discussion, the patient strongly expressed the desire and strongly opted for a vaginal delivery despite medical advice suggesting a cesarean section (C-section). The non-stress test (NST) indicated no signs of fetal distress (Figure 2).

**Figure 3:**
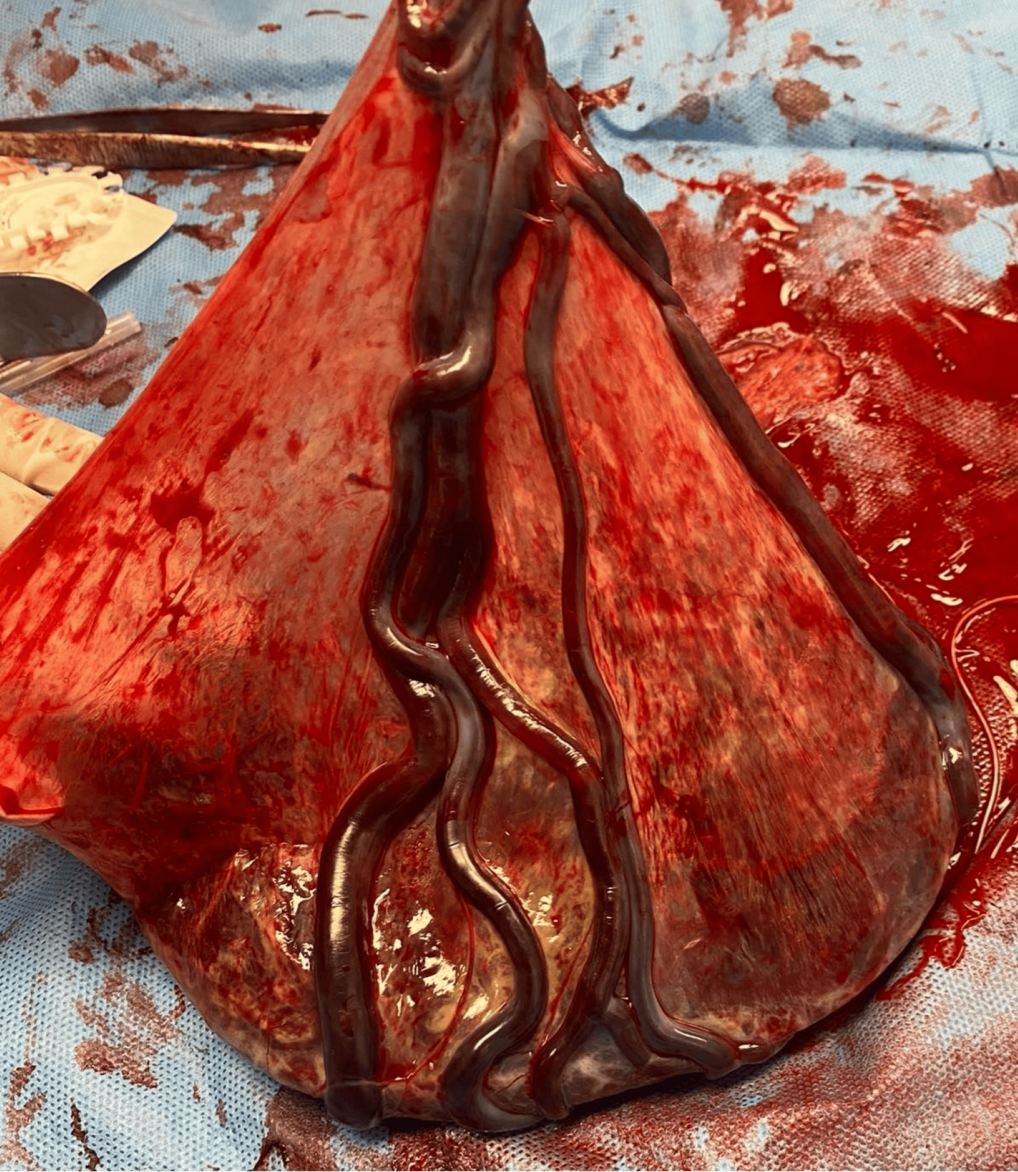
The gross examination of the placenta indicates a velamentous cord insertion, with the umbilical cord connecting to the fetal membranes rather than the placental disc.

No oxytocin IV or any other augmentation of labor was used, and after three hours, she gave birth to a female neonate weighing 2,770 g. The infant was delivered without any obstetric complications at 38 weeks and two days via vacuum-assisted vaginal delivery owing to uterine hypo-contractility at that point. On physical examination, VCI of the placenta was identified (Figure 3). 

There was no immediate postpartum hemorrhage. However, obstetric curettage was conducted for postpartum hemorrhage, and any vaginal and perineal lacerations were sutured. There were no cervical lacerations. Postpartum care included administration of methylergometrine three times daily for two days, ferrous sulfate of 800 mg/15 mL twice daily for one month, calcium of 500 mg twice daily for one month, and cefuroxime twice daily for three days following the uterine curettage. Both the neonate and the postnatal woman were discharged from the gynecology unit on the third day after admission. On the day of hospitalization, the day of discharge, and once more at 40 weeks, her test results were normal. Due to postpartum hemorrhage, a decrease in HBG was noted, particularly on the third day (Table 1).

**Table 1:** Laboratory results. WBC: white blood cell; LYMPH: lymphocyte; HGB: hemoglobin; HCT: hematocrit; INR: international normalized ratio; aPTT: activated partial thromboplastin time; PLT: platelet; CRP: C-reactive protein; AST: aspartate transferase; ALT: alanine transaminase; GGT: gamma-glutamyl transferase; ALB: albumin; GLC: glucose; TPR: total protein; UA: uric; URE: urea; CRE: creatinine; K+: potassium; Na+: sodium; TSH: Thyroid-stimulating hormone; FREE T4: free thyroxine

Parameter	Day 0 (admission day and labor day)	Day 1 (after labor)	Day 3 (exit day)	Follow-up 40 days (Postpartum period)	Reference Number
WBC	12.65 k/μL	19.28 k/μL	13.22 k/μL	11.65 k/μL	4-11 k/μL
Neutrophils	72.8%	88.9%	80.6%	66%	40-75%
LYMPH	18.4%	5.8%	13.8%	35%	20-45%
HBG	14.6 g/dL	10.6 g/dL	9.9 g/dL	13.5 g/dL	11.8-17.8 g/dL
HCT	39.9%	29.0%	28.0%	35.4%	36-52%
INR	0.86	Not taken	Not taken	Not taken	0.8-1.2
aPTT	23.93 sec	Not taken	Not taken	Not taken	26-36 sec
PLT	226 k/μL	178 k/μL	150 k/μL	320 k/μL	140-450 k/μL
CRP	0.66 mg/dL	1.02 mg/dL	0.80 mg/dL	0.20 mg/dL	0-0.80 mg/dL
AST	21 U/L	27 U/L	28 U/L	25 U/L	5-33 U/L
ALT	23 IU/L	18 IU/L	22 IU/L	24 IU/L	5-32 IU/L
GGT	21 IU/L	14 IU/L	25 IU/L	26 IU/L	5-31 IU/L
ALB	3.9 g/dL	3.0 g/dL	3.4 g/dL	3.2 g/dL	3.5-5.1 g/dL
GLC	106 mg/dL	92 mg/dL	95 mg/dL	85 mg/dL	70-115 mg/dL
TPR	6.2 g/dL	4.6 g/dL	4.8 g/dL	6.5 g/dL	6.2-8.4 g/dL
UA	4.3 mg/dL	4.5 mg/dL	4.0 mg/dL	2.5 mg/dL	2.3-6.1 mg/dL
URE	32 mg/dL	25 mg/dL	22 mg/dL	12 mg/dL	10-50 mg/dL
CRE	0.79 mg/dL	0.64 mg/dL	0.70 mg/dL	0.6 mg/dL	0.5-1.1 mg/dL
K+	3.9 mmol/L	4.0 mmol/L	3.8 mmol/L	3.6 mmol/L	3.5-5.1 mmol/L
Na+	137 mmol/L	136 mmol/L	139 mmol/L	140 mmol/L	136-146 mmol/L
TSH	2.3 mIU/L	Not taken	Not taken	2.0 mIU/L	0.3-3.0 mIU/L
FT4	13.2 pmol/L	Not taken	Not taken	1.,5 pmol/L	7.21-15.0 pmol/L

## Discussion

A case study involving a 36-week pregnant woman with a known history of furcate-velamentous umbilical cord insertion indicated that this type of cord insertion can lead to Benckiser's hemorrhage, a serious obstetric issue that carries a mortality risk for newborns between 75% and 100%. Timely identification of placental complications is essential in minimizing the risk of mortality by facilitating an urgent cesarean delivery [8].

A comprehensive study conducted in the Netherlands tracked 199 patients over a span of 17 years. The average age of the mothers was 33.6 years, while the average gestational age at delivery was 38.3 weeks. All participants underwent cesarean sections and had velamentous cord insertions. The research did not find any statistically significant difference in the rate of intrauterine fetal death between those with velamentous cord insertion and those without. Nonetheless, the incidence of intrauterine fetal death varied based on the timing and manner of the velamentous cord insertion across different groups [9]. According to a retrospective cohort analysis of nine patients with an average gestational week of 36.6, deliveries included three spontaneous vaginal, three instrumental, and four cesarean deliveries. Velamentous cord insertion was identified as a placental pathology. Among the fetal outcomes, two cases experienced intrapartum fetal bradycardia, while five had no complications. This study marks the first Doppler-based diagnosis of placental disease, which may enhance clinical decision-making and improve outcomes for newborns and postpartum women [10].

Case studies resulted in the findings that early diagnosis of velamentous cord insertion in the prenatal period, especially in the second trimester of pregnancy, can significantly reduce labor complications of VCI placenta pathology [11,12]. A retrospective cohort study involving 100 patients with VCI and 149 patients with marginal cord insertion (MCI) indicated that VCI was linked to a heightened risk of preterm delivery at 32-34 weeks. Additionally, there was no significant difference in the likelihood of preeclampsia or selective fetal growth restriction [13].

Retrospective analysis of 137 patients revealed that pregnant women with VCI delivered earlier than those with MCI. Additionally, comparisons reveal that birthweight was higher in the normal insertion group compared to those with VCI and MCI insertions [14]. Pregnant women with VCI face increased risks of preterm delivery, low birth weight, and small-for-gestational age compared to those with normal cord insertion, based on a 10-year observational study involving 501 cases of VCI and 59,976 normal cord insertions. The findings indicate a moderate risk of preterm birth, fetal growth retardation, and perinatal mortality associated with VCI [15].

Low risk factors, including a small fetus and multipara status, along with prompt management, can facilitate a safe vaginal delivery without serious complications, as illustrated in a case study of a 37-week pregnant woman with an undiagnosed history of VCI [16].

A retrospective case-control study involving 108 participants yielded no significant differences in second-trimester screening using uterine artery Doppler flowmetry (p-value = 0.001). The study found that factors linked to early preterm births in the VCI group included intrauterine hypoxia, spontaneous premature rupture of membranes, premature labor, malformation-related terminations, and intrauterine fetal demise. The rate of cesarean sections remained unchanged. Furthermore, intrauterine death and fetal abnormalities were significantly more prevalent in VCI pregnancies (p-value: < 0.001), with notable malformations, including congenital cardiac diseases, gastrointestinal issues, thoracic anomalies, central nervous system defects, and facial or skeletal deformities [17]. All pertinent research is referenced in Table 2.

**Table 2:** Relevant studies on VCI placenta pathology in pregnancy women. VCI: velamentous cord insertion; SUA: single umbilical artery; MCI: marginal cord insertion

Author/Publication Year/Reference Number	Type of Study	Number of Patients	Maternal Age Median (years)	Gestational Weeks	Delivery Type	Placenta Pathology	Pregnancy Complications	Fetal Outcomes	Results
Papasavva et al. (2025) [8]	Case Report	1	Not mentioned	36	Cesarean delivery	Furcate-velamentous insertion of the umbilical cord	Metrorrhagia and decreased fetal movements	Hypoxic-ischemic encephalopathy	VCI can result in Benckiser's hemorrhage, a grave obstetric complication that poses a mortality risk to newborns ranging from 75% to 100%. Early detection of placental issues is crucial in reducing this mortality risk through prompt cesarean section
Koorn et al. (2025) [9]	Retrospective Case-Control Study	199	33.6	38.3	Cesarean delivery	VCI	Location of insertion: anterior (n=24), posterior (n=17), lateral (n=45). Intrauterine fetal death (n=1)	Fetal growth restriction (n=46), p-value: <0.001	No statistically significant difference was found between cases of intrauterine fetal death and velamentous cord insertion, although there is variation in the rates of intrauterine fetal death depending on the timing and method of velamentous cord insertion across different groups
Moral-Moral et al. (2024) [10]	Retrospective Cohort Study of Case Series	9	not mentioned	36.6	Spontaneous vaginal delivery: 3 cases (33.3%), instrumental delivery: 2 cases (22.2%), cesarean section: 4 cases (44.4%)	VCI	Isolated succenturiate placenta: 5 cases (55.6%), succenturiate placenta and VCI: 4 cases (44.4%), succenturiate placenta and vasa previa: 0 cases (0%)	Intrapartum fetal bradycardia: 2 cases (22.2%), placenta abruption: 1 case (11.1%), threatened preterm labor: 1 case (11.1%); no complications: 5 cases (55.6%)	The need and use of prenatal identification of placental pathology via Doppler is crucial for accurate clinical decisions and thereby improving outcomes for postnatal women and newborn infants
Potosí-García et al. (2024) [11]	Case Report and Review of the Literature	1	27	38.5	Cesarean delivery	VCI	No complications reported	No complications reported	Early diagnosis and management of VCI can significantly reduce labor complications
Inatomi et al. (2024) [12]	Case Report and Review of the Literature	1	38	40	Cesarean delivery	VCI and SUA	Massive vaginal bleeding	Fetal bradycardia	Monitoring during the second trimester of pregnancy aids obstetric clinicians in preparing for urgent treatment of women facing severe placental pathologies
Li Wen et al. (2023) [13]	Retrospective Cohort Study	VCI: 100 Marginal cord insertion (MCI): 149	not mentioned	VCI: 36, MCI: 38.3	Not mentioned	VCI and MCI	No risk for preeclampsia	No result for fetal growth restriction and small-for-gestational-age fetus	Pregnancies with VCI are at a higher risk of preterm delivery at 32-34 weeks; however, there is no significant difference in the risk of preeclampsia or selective fetal growth restriction
Gu et al. (2022) [14]	Retrospective study	137	36	Normal cord insertion: 57, MCI: 34, VCI: 46	Not mentioned	VCI and MCI	No complications reported	No complications reported	VCI gestational age at delivery was significantly earlier (p-value: < 0.05), while monochorionic diamniotic twins were not significantly affected by placenta pathologies such as VCI and MCI (p-value: > 0.05)
Yang et al. (2019) [15]	Observational study	Normal cord insertion: 59.475, VCI: 501	VCI: 31, Normal cord insertion: 33	VCI: 38.4 and Normal cord insertion: 39.4	Cesarean delivery and Forceps delivery	VCI	Rupture of vasa previa: 1 (14.3%), exaggerated torsion of umbilical cord: 1 (14.3%), prolapse of umbilical cord: 1 (14.3%), and placenta abruption: 1 (14.3%), vaginal hemorrhage	Perinatal deaths: 7, low birth weight: 3	Lower gestational age in a woman with VCI (p-value < 0.001), selective and emergent cesarean delivery in women who had VCI than those who had normal cord insertion (p-value: < 0.001)
Li et al. (2018) [16]	Case Report	1	31	37	Vaginal delivery	VCI	Vaginal hemorrhage	No complications reported	Low risk factors may contribute to having a natural vaginal delivery without postnatal women and newborn infants
Yerlikaya et al. (2015) [17]	Retrospective Case-Control Study	108	31.6	37	Cesarean delivery	VCI	Cases with VCI revealed the last measurement of the umbilical artery before delivery was 1.00 ± 0.25 (p-value: = 0.001). There was no difference in the measurements at the gestational age	Fetal abnormalities and intrauterine demise were considerably more prevalent in pregnancies involving VCI (12.7 vs 0%) (p-value: < 0.001)	Pregnancies with VCI were linked to higher rates of early preterm delivery, anomalies, and intrauterine fetal death (IUFD)

A meta-analysis compared MCI, VCI, and normal placenta cord insertion (PCI) and found that both MCI and VCI (pooled relative risk (RR): 2.86, 95% confidence interval (CI): 1.56-5.22, p-value: 0.0006) and PCI (pooled RR: 1.77, 95% CI: 1.33-2.36, p-value: < 0.0001) were associated with a statistically significant increased risk of requiring emergency cesarean delivery [18].

Another study that performed a meta-analysis found that women with VCI are at higher risk of small-for-gestational-age neonates (RR: 2.69; 95% CI: 1.73-4.17), pregnancy-induced hypertension (RR: 1.94; 95% CI: 1.24-3.01), and stillbirth (RR: 9.42; 95% CI: 3.19-27.76), but not preterm delivery (RR: 1.92; 95% CI: 0.82-4.54) [19].

This case study presents a postnatal woman diagnosed with VCI who underwent a vaginal delivery without complications for both the infant and the postpartum woman. The literature indicates that numerous cases and research articles suggest that this pathology may influence the fetus, although the mechanisms of its development and potential effects on the fetus and postpartum woman remain unclear. Further cohort studies and biochemical research focusing on placentas are necessary to gain a deeper understanding of this severe placental pathology and clinicians’ decisions on how to manage pregnant women who suffer from VCI during pregnancy.

## Conclusions

VCI, arising from inadequate Wharton's jelly surrounding the umbilical vasculature, poses significant risks to the fetus, including compression, hypoxia, and potential hemorrhage. Effective risk assessment, counseling, and birth planning hinge on early and accurate prenatal diagnosis through color Doppler imaging and detailed ultrasonography during the whole gestation. Although cesarean sections are commonly recommended to mitigate intrapartum risks, successful vaginal birth can be achieved in carefully selected cases, provided that continuous fetal monitoring is in place and no signs of fetal impairment are present. Optimizing obstetric care necessitates a personalized approach that considers maternal preferences and timely interventions. Current evidence suggests that vaginal delivery may not be universally contraindicated solely due to velamentous cord insertion. To establish consistent guidelines for the optimal delivery method in cases involving this condition, further comprehensive research is essential. Future medical professionals are urged to use patient-centered, evidence-based strategies rather than reflexive intervention. VCI alone should not automatically prevent a planned vaginal birth when fetal well-being is guaranteed, and continuous intrapartum monitoring is available, even if cesarean delivery may be needed in high-risk situations. The ultimate objective is to provide accurate, compassionate treatment that is sensitive to the distinct clinical situation of each pregnancy, rather than just managing risk.
